# A Glyphosate-Based Herbicide in Soil Differentially Affects Hormonal Homeostasis and Performance of Non-target Crop Plants

**DOI:** 10.3389/fpls.2021.787958

**Published:** 2022-01-27

**Authors:** Benjamin Fuchs, Miika Laihonen, Anne Muola, Kari Saikkonen, Petre I. Dobrev, Radomira Vankova, Marjo Helander

**Affiliations:** ^1^Biodiversity Unit, University of Turku, Turku, Finland; ^2^Laboratory of Hormonal Regulations in Plants, Institute of Experimental Botany of the Czech Academy of Sciences, Prague, Czechia; ^3^Department of Biology, University of Turku, Turku, Finland

**Keywords:** environmental pollutants, cascading herbicide effects, plant ecology, plant defense, shikimate pathway, plant physiological regulation

## Abstract

Glyphosate is the most widely used herbicide with a yearly increase in global application. Recent studies report glyphosate residues from diverse habitats globally where the effect on non-target plants are still to be explored. Glyphosate disrupts the shikimate pathway which is the basis for several plant metabolites. The central role of phytohormones in regulating plant growth and responses to abiotic and biotic environment has been ignored in studies examining the effects of glyphosate residues on plant performance and trophic interactions. We studied interactive effects of glyphosate-based herbicide (GBH) residues and phosphate fertilizer in soil on the content of main phytohormones, their precursors and metabolites, as well as on plant performance and herbivore damage, in three plant species, oat (*Avena sativa*), potato (*Solanum tuberosum*), and strawberry (*Fragaria x ananassa*). Plant hormonal responses to GBH residues were highly species-specific. Potato responded to GBH soil treatment with an increase in stress-related phytohormones abscisic acid (ABA), indole-3-acetic acid (IAA), and jasmonic acid (JA) but a decrease in cytokinin (CK) ribosides and cytokinin-O-glycosides. GBH residues in combination with phosphate in soil increased aboveground biomass of potato plants and the concentration of the auxin phenylacetic acid (PAA) but decreased phaseic acid and cytokinin ribosides (CKR) and O-glycosides. Chorismate-derived compounds [IAA, PAA and benzoic acid (BzA)] as well as herbivore damage decreased in oat, when growing in GBH-treated soil but concentrations of the cytokinin dihydrozeatin (DZ) and CKR increased. In strawberry plants, phosphate treatment was associated with an elevation of auxin (IAA) and the CK *trans*-zeatin (tZ), while decreasing concentrations of the auxin PAA and CK DZ was observed in the case of GBH treatment. Our results demonstrate that ubiquitous herbicide residues have multifaceted consequences by modulating the hormonal equilibrium of plants, which can have cascading effects on trophic interactions.

## Introduction

Glyphosate-based herbicides (GBH) are the world’s most widely used pesticides due to their affordable price and efficiency in killing weeds (Myers et al., 2016). The rise in GBH application rate has increased the contamination hazard of glyphosate residues and degradation products in crop habitats globally (Maggi et al., 2020; Muola et al., 2021). Glyphosate targets 5-enolpyruvylshikimate-3-phosphate synthase (EPSPS), the key enzyme in the shikimate pathway present in plants (Duke and Powles, 2008). By inactivating this enzyme, glyphosate interrupts the supply of chorismate which is the essential precursor for the biosynthesis of three aromatic amino acids tryptophan, phenylalanine and tyrosine (Maeda and Dudareva, 2012). Chorismate and these amino acids are key components of numerous primary and secondary compounds including the phytohormones indole-3-acetic acid (IAA) and salicylic acid (SA) which are involved in plant stress responses to many abiotic and biotic stressors (Kazan and Manners, 2009; Rekhter et al., 2019; Ding and Ding, 2020). Furthermore, many other organic compounds, such as the diverse group of phenylpropanoids, are derived from the shikimate pathway and shown to be affected by low glyphosate doses (Ossipov et al., 2003; Zabalza et al., 2017). Phenylpropanoids are essential compounds in plant interactions with various organisms from beneficial microorganisms and insect pollinators to herbivores and pathogens (Deng and Lu, 2017). Thus glyphosate residues may indirectly alter plant interactions by affecting core biochemical pathways (Fuchs et al., 2021).

Recently, the presence of glyphosate residues and degradation products have been confirmed from diverse agricultural habitats all across the planet, which has drawn the attention to potential effects of these residues in soil on the biology and ecology of organisms (Maggi et al., 2020; Fuchs et al., 2021). Glyphosate is proclaimed to pose negligible ecological risks because it is assumed to be degraded quickly by soil microorganisms into aminomethylphosphonic acid (AMPA) and sucrose (Borggaard and Gimsing, 2008). However, accumulating evidence shows that the rate of glyphosate degradation depends on environmental conditions and may be slow, especially in colder climates (Helander et al., 2012, 2018). In addition, glyphosate residues have been shown to affect diverse soil organisms which in turn can affect plants and their interactions with the environment (Aristilde et al., 2017; Helander et al., 2018, 2019; Niemeyer et al., 2018; Rainio et al., 2020). Experiments with low glyphosate doses showed physiological changes in plants, such as altered photosynthesis (Gomes et al., 2014, 2017; Serra et al., 2015; Soares et al., 2020), oxidative stress (Spormann et al., 2019), tannin biosynthesis (Ossipov et al., 2003) or change of plant volatile composition (D’Alessandro et al., 2006). However, it is unknown how residues of GBH in soil affect core plant physiological processes, such as phytohormone biosynthesis. Phytohormones regulate plant developmental processes as well as their interactions with the biotic environment ranging from antagonistic pathogens and herbivores to mutualistic microbes and beneficial insects (Robert-Seilaniantz et al., 2011; Gruden et al., 2020).

The aim of our study was to determine the effect of GBH residues in soil on phytohormone pools of three different crop species. Phytohormones regulate plant growth and development as well as responses to abiotic and biotic environment (Berens et al., 2017; Liu et al., 2020). Furthermore, phytohormones build a signaling network and they may interact synergistically or antagonistically with each other, which may modulate the outcome of physiological responses in plants to biotic and abiotic stimuli in an unexpected manner, such as responses to herbivores, which are often mediated by phytohormones (Robert-Seilaniantz et al., 2011; Munné-Bosch and Müller, 2013; Berens et al., 2017; Gruden et al., 2020; Fuchs et al., 2021). Thus, we monitored plant performance and herbivore damage.

In this study, we performed extensive phytohormone profiling in leaves of garden strawberry (*Fragaria x ananassa*), oat (*Avena sativa*), and potato (*Solanum tuberosum*) grown in soils which were treated with GBH according to common agricultural practices for six consecutive years. Phosphate application was included into the study because it competes with glyphosate for binding sites in soil particles, and thus plays an important role in determining the availability of glyphosate to plants (Wang et al., 2005; Padilla and Selim, 2019). The affinity of phosphate to soil particles is higher compared to glyphosate, which facilitates both, the bioavailability of glyphosate to the plant and at the same time, the possibility of leaching from treated areas, depending on i.e., soil properties and precipitation (Borggaard and Gimsing, 2008).

We hypothesize that GBH residues in soil (1) decrease concentrations of phytohormones, which biosynthesis is based on products from the shikimate pathway, (2) have a stronger effect on phytohormone concentrations after phosphate addition due to the increased bioavailability, (3) differentially affect plant species depending on their physiology and life history strategy, and (4) increase herbivore damage due to a disrupted hormone equilibrium.

## Materials and Methods

### Study Species and Experimental Design

Three crop plant species were chosen on the basis of their (i) horticultural and agronomical importance, (ii) contrasting physiology, and (iii) plant life history strategy, which may affect the physiological susceptibility to glyphosate residues in soils. Strawberry represents perennial dicots of the family Rosaceae. They reproduce *via* aboveground organs, either sexually (*via* fruiting) or asexually (*via* runners). Oat is an annual monocot as many cultivated cereal plants globally. It belongs to the Poaceae family, which covers many forage grasses. Potato belongs to the plant family Solanaceae. It is a perennial plant with belowground storage/reproductive organs, which are in direct contact with the soil and GBH residues.

The experiment was conducted on a field site at Ruissalo Botanical garden (60°26′N, 22°10′E) in southwestern Finland in 2019. The field consisted of ten alternating GBH (10 replicates) and ten control (10 replicates) plots (each plot had a dimension of 23 m × 1.5 m), separated by 0.5 m buffer zones to avoid possible contamination of control plots with GBH. The soil is medium clay with high organic matter and a generally high phosphate affinity. Physicochemical properties of the soil were generally assessed and the pH ranged between 6.0 and 6.5. Furthermore we assessed levels of Calcium (between 2100 and 2600 mg l^–1^), Phosphorus (between 4.0 and 6.0 mg l^–1^), Potassium (between 200 and 250 mg l^–1^), Magnesium (between 450 and 550 mg l^–1^) and Sulfur (between 16 and 22 mg l^–1^).^1^ The experimental field has been treated according to the following description, since 2014. The experimental setup and field conditions are outlined in more detail in Helander et al. (2019). After tilling, GBH plots were sprayed with Roundup Gold (glyphosate concentration 450 g L^–1^, CAS: 3864-194-0, application rate: 6.4 L ha^–1^, in 3 L of tap water per plot, applied on May 13, 2019) on a windless day with a hand operated pressure tank using a plastic hood in the sprinkler tip, to prevent the glyphosate from spreading outside the treatment plots. 16.2 ml of Roundup Gold were diluted in 3 L tab water and homogenously sprayed per plot which equals to ∼7.2 g of glyphosate per plot. Control plots were sprayed only with water (3 L of tap water per plot). We used a dosage of glyphosate of 3 kg ha^–1^ which is within the field realistic doses. Before GBH and water treatment, each plot was divided in half and the resulting alternating subplots were treated either with phosphate (P) fertilizer (Yara Ferticare 80 g in 10 L of water per subplot, applied between 29 April and 3 May) or equivalent amount of water (control subplots). Our experimental setup resulted in four treatment groups: phosphate + GBH (PG), GBH only (G), phosphate only (P), control (C).

We sowed 30 oat seeds, planted 6 potato tubers and 6 young strawberry plantlets on each of the ten subplots per treatment 24 days after the GBH application on 6 June, which exceeds the recommended safety period of the used product.

### Data Collection in the Field

For phytohormone analysis, we sampled one randomly chosen oat, potato and strawberry plant from each subplot (altogether 80 samples). On 6 August, 63 days after the planting, we cut ∼200 mg of comparable fresh leaf material from each plant. Leaf samples were immediately placed into a 2 mL reaction tube (Eppendorf GmbH) and flash frozen in liquid nitrogen. Samples were kept at −80°C until hormone analyses.

We analyzed the effect of GBH and phosphate fertilizer on plant damage caused by naturally occurring herbivores on all three crop species on 30 July (potato), 1 August (oat, 5 perceptually median individuals), and 15 August (strawberry). To estimate herbivore damage, we assessed the total amount of damage each plant had experienced (i.e., damage by different herbivores was not assessed separately). Only leaf damage by feeding was quantified. According to the amount of damage, we assigned plants into four classes similar to previous studies (Poveda et al., 2010): 0 no signs of herbivory, 1 up to 10%, 2 up to 30%, and 3 up to 50% of leaf area damaged. Potato plant showed almost no signs of damage, probably due to a lack of specialized herbivores.

As a measure of potato size, we recorded plant height, number of stems, the fresh weight of tubers and dry weight of aboveground plant parts at the end of the experiment (20–23 August). Strawberry size was measured by counting the number of fully developed leaves. Oat biomass was not determined due to an advanced stage of wilting throughout the individuals in August.

### Glyphosate Residue Analysis

We monitored the degradation and persistence of glyphosate by taking soil samples in a late stage of the experiment on 15 August. Soil samples were taken from each subplot and samples were pooled. Each soil sample was ∼2.5 cm in diameter and 5 cm in depth. Sampled soil material was air-dried before analysis for glyphosate and its degradation product AMPA. Extraction was performed with aqueous, acidified methanol followed by analysis *via* liquid chromatography coupled to mass spectrometry (LC-MS/MS) at Groen Agro.^2^

### Hormone Extraction and Quantification

Frozen samples (ca 200 mg FW) were homogenized with liquid nitrogen in mortar and pestle and weighed. Phytohormones were extracted with cold (−20 °C) methanol/water/formic acid (15/4/1, v/v/v) as described in Dobrev and Vankova (2012) and Djilianov et al. (2013). The following isotope-labeled internal standards (10 pmol per sample) were added: ^13^C_6_-IAA, ^2^H_2_-OxIAA (Cambridge Isotope Laboratories); ^2^H_4_-SA [Sigma-Aldrich); ^2^H_3_-PA, ^2^H_3_-DPA, ^2^H_4_-7OH-ABA, ^2^H_5_-ABA-GE (NRC-PBI); ^2^H_6_-ABA, ^2^H_5_-JA, ^2^H_5_-tZ, ^2^H_5_-tZR, ^2^H_5_-tZRMP, ^2^H_5_-tZ7G, ^2^H_5_-tZ9G, ^2^H_5_-tZOG, ^2^H_5_-tZROG, ^2^H_3_-DZ, ^ 2^H_3_-DZR, ^2^H_3_-DZ9G, ^2^H_3_-DZRMP, ^2^H_7_-DZOG, ^2^H_6_-iP, ^2^H_6_-iPR, ^2^H_6_-iP7G, ^2^H_6_-iP9G, ^ 2^H_6_-iPRMP (Olchemim)]. The extract was centrifuged (17,000 × *g*, 4 °C, 20 min) to remove solid debris. It was then concentrated using an Alpha RVC vacuum centrifuge (Christ; 40 °C, 15 mbar, 1.5 h). Phytohormones were separated with a reverse-phase–cation exchange SPE column (Oasis-MCX, Waters) into the acid fraction by elution with methanol (auxins, ABA, SA, JA), and into the basic fraction by elution with 0.35 M NH_4_OH in 60% methanol [cytokinins (CKs), 1-Aminocyclopropane-1-carboxylic acid (ACC]. Fractions were dried in the vacuum centrifuge and resuspended in 30 μl acetonitrile (15%) in the case of acid fraction and in 5% methanol in the case of basic fraction. Hormones were analyzed using HPLC (Ultimate 3000, Dionex) coupled to 3200 Q TRAP hybrid triple quadrupole/linear ion trap mass spectrometer (Applied Biosystems). Hormone quantification was carried out using the isotope dilution method with multilevel calibration curves (*r*^2^ > 0.99). Data were processed with the Analyst 1.5 software package (Applied Biosystems).

### Statistical Analyses

Hormone concentrations, plant height (potato), biomass (potato) and leaf numbers (strawberry) were analyzed with ANOVA and Dunnett’s *Post hoc* test, which tests for differences between all treatments (G, P, and PG) and the control. To account for the potential environmental variation between the different parts of the experimental field, we included plot as a random factor, which was not significant in any of the analysis. Plant herbivore damage categories/classes were compared with Chi-squared test followed by a Fisher‘s exact test, testing the proportions of each damage category within the four treatments against each other. Selected hormone concentrations were correlated with Pearson product moment correlation to indicate their interaction or biosynthetic connection. All statistical analyses were conducted using the open-source software R (R version 3.6.1). Graphical illustrations were conducted with the R package ggplot2 (Wickham, 2016) and the use of the software ChemDraw (version 19.1, PerkinElmer Informatics, Inc.).

## Results

### Glyphosate Residues in Soils of Different Treatments

Glyphosate and its degradation product AMPA residues were markedly higher in GBH-treated soils compared to control (C) soils, irrespective of phosphate (P) treatment. We detected 60 and 70 μg kg^–1^ of glyphosate in samples from GBH (G) and phosphate and GBH (PG) treated soils, respectively. AMPA concentrations in soils from G and PG treatments were 1.9 and 1.8 mg kg^–1^, respectively. In contrast, both glyphosate and AMPA concentrations were below quantification limit (concentrations ≤0.01 mg kg^–1^ for glyphosate and ≤0.05 mg kg^–1^ for AMPA) in soil samples collected from C and P treatments.

### Plant Performance and Phytohormone Pools in Oat

**FIGURE 1:**
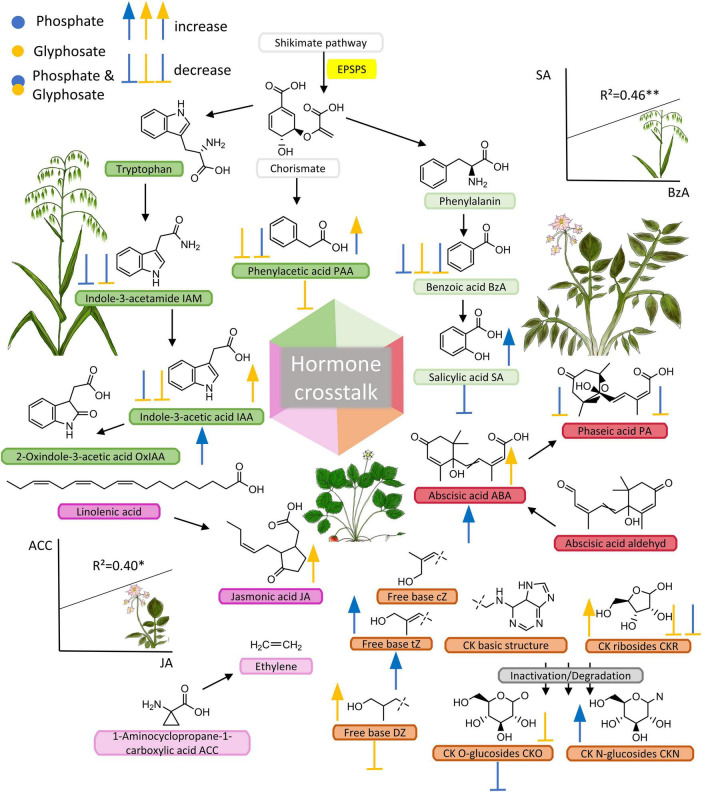
Phytohormone pools affected by GBH, phosphate fertilizer and their combination in three crop species. Phytohormones, their precursors and metabolites; compounds that were analyzed in this study are indicated with abbreviations after the compound name. Additional compounds (without abbreviations) were added to show common pathway intermediates and to show biosynthetic origins of phytohormones. Chorismate derives from the shikimate pathway which includes the target site of glyphosate. By blocking the EPSPS enzyme, an essential biosynthetic step is corrupted, which is often shown to cause decreased biosynthesis of metabolites synthesized downstream of the shikimate pathway. Centrally placed hexahedron highlights the possible interactions between hormones also known as hormone crosstalk. Symbols (arrows and stops) besides and below metabolites (left side = oat, right = potato, and below = strawberry) indicate the effect of treatment (blue = phosphate, yellow = GBH, blue + yellow = phosphate + GBH) on each plant species corresponding to significances shown in Figure 2 (*N* = 40).

**FIGURE 2:**
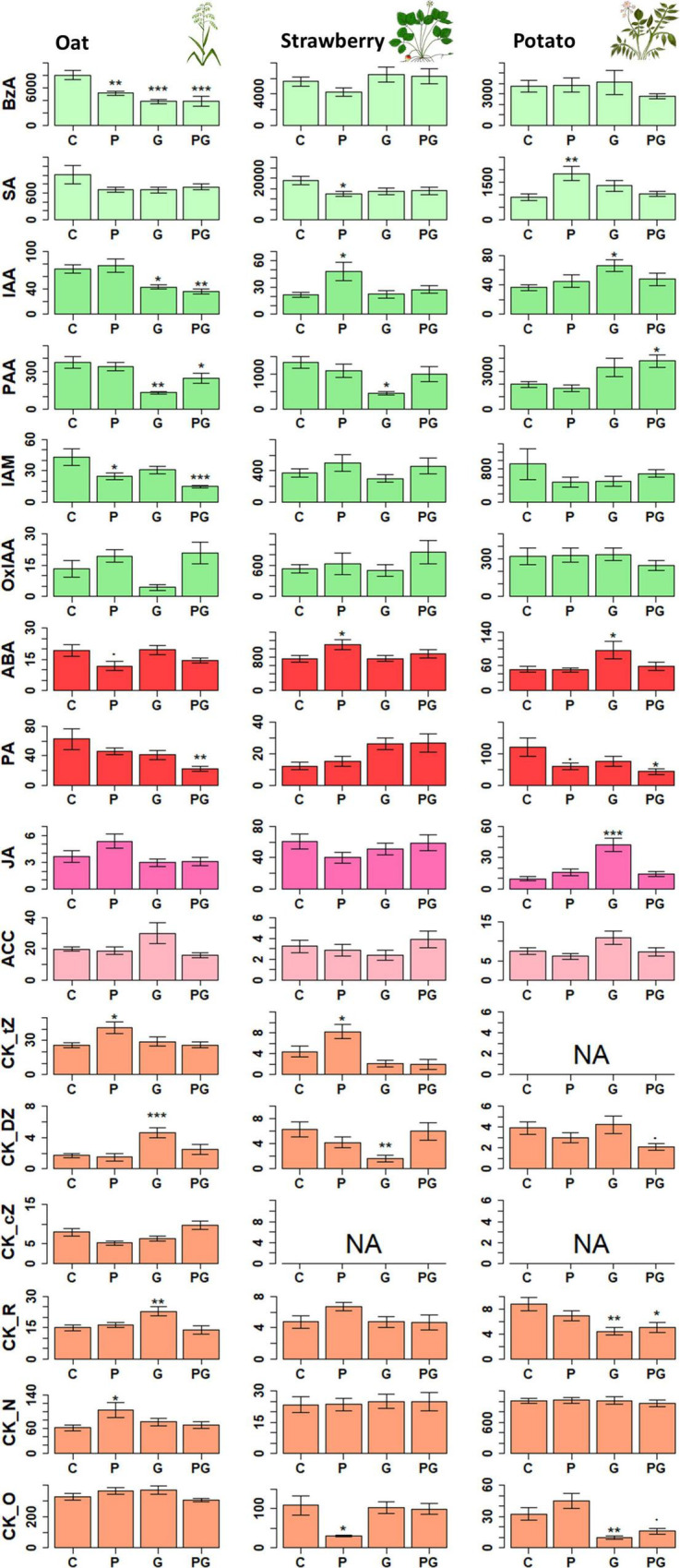
The effect of different treatments (P, G, and PG) on the leaf phytohormone levels in oat, potato and strawberry. Statistical significance of differences in phytohormone concentrations between control and individual treatments was tested with Dunnett’s *post hoc* test following ANOVA (detailed results see Supplementary Table 1 for mean ± S.E values and Supplementary Table 2 for F and *p* values). *p < 0.05, **p < 0.01, and ***p < 0.005. NA data not available due to concentrations below detection limit. Pathway affiliation of each compound is presented in Figure 1. Concentrations are given in pmol g^–1^ fresh weight. *N* = 40. BzA, benzoic acid; SA, salicylic acid; PAA, phenylacetic acid; IAM, indole-3-acetamide; IAA, indole-3-acetic acid; OxIAA, 2-Oxindole-3-acetic acid; ABA, abscisic acid; PA, phaseic acid; JA, jasmonic acid; ACC, 1-Aminocyclopropane-1-carboxylic acid; CK, cytokinin; DZ, dihydrozeatin; tZ, *trans*-zeatin; cZ, cis-zeatin; _R, ribosides; _O, O-glycosides; _N, N-glycosides.

The concentration of benzoic acid (BzA), an intermediate product of shikimate pathway involved in biosynthesis of many secondary metabolites, was suppressed by all treatments compared to control in oat (Figures 1, 2 and Supplementary Table 2). Similar to BzA, the phytohormone SA, also one of the products of this pathway, was slightly, but not significantly suppressed in the treatments compared to control. Pearson correlation test confirmed a linear relationship between BzA and SA compounds (*r*^2^ = 0.46, *df* = 38, *p* = 0.003, Figure 1) indicating their biosynthetic dependency in oat (Lefevere et al., 2020). Concentration of the auxins IAA and phenylacetic acid (PAA) were lower in oat leaves from GBH treatment alone as well as in GBH + phosphate treatment compared to control (Figures 1, 2 and Supplementary Table 2). Similarly, GBH treatment alone, and even stronger in combination with phosphate, suppressed the level of the IAA precursor indole-3-acetamide (IAM) (Figures 1, 2 and Supplementary Table 2). GBH treatment increased the levels of the active CK base dihydrozeatin (DZ), while phosphate caused the elevation of *trans*-zeatin (tZ) (Figures 1, 2 and Supplementary Table 2), which was accompanied by increase of CK deactivation metabolites CK N- glucosides (CKN) (Figures 1, 2 and Supplementary Table 2). Cytokinin ribosides (CKR) were significantly elevated by GBH treatment (Figures 1, 2 and Supplementary Table 2). Herbivore damage was lower in oat plants from GBH treatment (Table 1). None of the oat plants showed herbivory damage higher than 50% of leaf area.

**TABLE 1:** The effect of different treatments (P, G, and PG) on leaf damage on oat and strawberry.

*Oat*	X-squared	*df*	*p*-value	Damage (%)			
				C	P	G	PG
Class 0	0.263	3	0.967	19.4	18.6	18.6	17.3
Class 1	0.504	3	0.918	51.7	51.7	52.5	49.1
Class 2	2.527	3	0.470	19.4	19.3	26.0	22.9
Class 3	10.878	3	0.012*	9.5	10.4	2.9*	11.7
***Strawberry***							
Class 0	0.932	3	0.817	25.9	21.1	27.6	20.3
Class 1	0.686	3	0.876	77.8	71.9	65.5	67.8
Class 2	2.499	3	0.476	14.8	7.02	6.9	11.9
Class 3	/	/	/				

Leaf damage was quantified by assigning plants to four different categories according to the amount of damage: class 0 no signs of leaf damage, 1 up to 10% of leaf area damaged, 2 up to 30% of leaf area damaged, and 3 up to 50% of leaf area damaged. None of the oat plants had more than 50% of leaf area damaged, and none of the strawberry plants had more than 30% of leaf area damaged. Strong leaf damage (Class 3) occurred less frequent in oat plants of the G treatment compared to plants from other treatments; analyzed with Chi-squared test for given probabilities and Fisher‘s exact test for pairwise comparisons. Significant results are marked with *p < 0.05 (N = 576).

### Plant Performance and Phytohormone Pools in Potato

**FIGURE 3:**
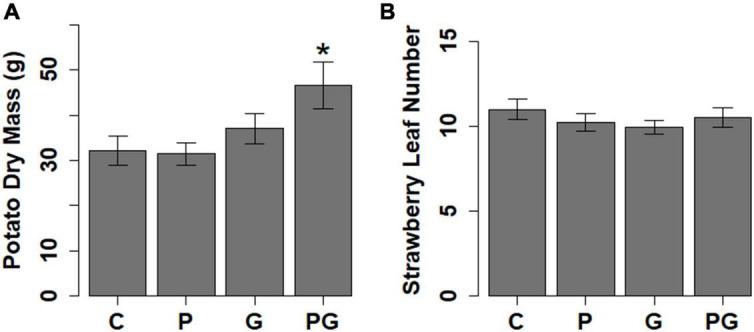
The effect of different treatments (C, control; P, phosphate fertilizer; G, GBH application and PG, phosphate fertilizer and GBH application) on **(A)** potato growth measured as aboveground biomass and **(B)** strawberry growth measured as number of leaves. Statistical significance between control and different treatments was tested with Dunnett’s *Post hoc* test following significant ANOVA, *p < 0.05 (*N* = 128 for potato, *N* = 131 for strawberry).

Potato responded to GBH residues in soil by increasing the concentration of abscisic acid (ABA), jasmonic acid (JA), and IAA (Figures 1, 2 and Supplementary Table 2). The other analyzed auxin, PAA, was up-regulated in combined GBH and phosphate treatment (Figures 1, 2 and Supplementary Table 2). Ethylene precursor (ACC) was slightly but non-significantly elevated in samples collected from GBH treatment and it positively correlated with JA concentration (*r*^2^ = 0.41, *df* = 36, *p*-value = 0.01, Figure 1). CKR were decreased in GBH treatment as well as in the combined GBH and phosphate treatment. Similar response was found in the case of CK deactivation metabolites CK O-glucosides (CKO) (Figures 1, 2 and Supplementary Table 2). SA significantly increased in potato only in response to phosphate and there was no significant correlation with BzA, indicating a BzA-independent biosynthesis of SA. Plant height and aboveground plant biomass were higher for potatoes growing in GBH + phosphate treatment (Figure 3A). Herbivory damage of potato plants was not evaluated due to very low incidences in general.

### Plant Performance and Phytohormone Pools in Strawberry

Glyphosate-based herbicide treatment caused a decrease in the concentration of the auxin PAA and the CK base DZ (Figures 1, 2 and Supplementary Table 2). The combined GBH and phosphate treatment did not significantly alter phytohormone levels in strawberry plants. However, phosphate treatment alone caused an increase of the phytohormones IAA, ABA and the most physiologically active CK tZ. Simultaneously, a decrease in SA and the inactive CK metabolites CKO was observed (Figures 1, 2 and Supplementary Table 2). Neither strawberry size nor amount of herbivory differed between the treatments (Figure 3B and Table 1). Strawberry leaf damage did not exceed 30% of leaf area.

## Discussion

Our results show that the residues of GBH in soil modulated plant hormonal homeostasis, plant size and herbivore damage. However, results varied among studied crop species and depended on phosphate fertilization. Overall, responses to GBH residues were strongest in potato (*Solanum tuberosum*) where we recorded increased levels of stress-related phytohormones ABA, JA, and SA. Hormonal responses in oat (*Avena sativa*) differed distinctly from potato and strawberry (*Fragaria x ananassa*), confirming that GBH residues in soil causes considerable differences between plant species. This may be caused by species specific architecture and morphology but also plant life-history strategies may partly explain the detected differences among the species representing different clades and survival/reproduction strategies. As a representative of annual monocot Poaceae, oat is rather adapted to invest resources into seed production than storage in leaves or defense during the course of its relatively short lifespan (Hodgson et al., 2020). In contrast, strawberry plants don’t necessarily flower during their first growing season but invest resources primarily into vegetative growth. Potatoes allocate substantial amount of resources to storage organs in roots which may explain the relatively strong hormone response.

### Phytohormones Derived From the Shikimate Pathway

Confirming our hypothesis that GBH residues in soil decrease the biosynthesis of phytohormones relying on aromatic amino acids as precursors by inhibition of EPSPS (Sharon et al., 1992), we detected decreased levels of IAA, auxin PAA, and BzA which is a precursor of SA in oats growing in GBH-treated soil (Lefevere et al., 2020). Similarly, GBH residues in soil reduced the production of the auxin PAA in strawberry, albeit the reduction was rather weak compared to that observed in oat. Decreased IAA concentrations has been recorded in tobacco (*Nicotiana tabacum*), when pretreated with glyphosate (Lee et al., 1983). In contrast, the production of IAA, PAA, and SA were up-regulated in potato plants demonstrating the complexity of physiological processes involving compounds derived from the shikimate pathway when challenged with GBH residues in soil. In glyphosate-resistant cotton glyphosate caused an increase in IAA concentrations due to an inhibition of auxin transporters (Yasuor et al., 2006). However, it is likely that decreased concentrations of shikimate-derived phytohormones in oat plants reflect the direct inhibitory effect which glyphosate has on the EPSPS enzyme (Hoagland, 1980), while the increased levels of phytohormones in potato may reflect stress-mediated mobilization of hormones involved in plant defense (Kahl, 1974).

### Phytohormones Produced Independent of the Shikimate Pathway

In addition to phytohormones with a biosynthetic link to the shikimate pathway, GBH residues in soil modulated the biosynthesis of several other examined phytohormones. For example, the production of the active CK DZ was increased in oat but decreased in strawberry, and ABA increased in potato. CKs are commonly involved in growth regulation and ABA is commonly perceived as stress hormones, but both are known to be induced when plants are subjected to abiotic stressors (Asselbergh et al., 2007; Havlová et al., 2008). These results indicate that GBH residues in soil can affect the production of different stress-related hormones in different plants even though these hormones are not directly linked to the shikimate pathway. The regulation of phytohormones can further be indirectly affected *via* hormone crosstalk with other phytohormones but the role of glyphosate, its breakdown products and additives in GBH are still unclear (indicated in Figure 1; Robert-Seilaniantz et al., 2011; Gomes et al., 2014). One example is ethylene, which can act synergistically with JA (Adie et al., 2007), as indicated by the positive correlation between the concentrations of the ethylene precursor ACC and JA in potato (Figure 1). Exogenous ACC treatment has been linked to increased JA-mediated biochemical processes in lima bean (Horiuchi et al., 2001). Ethylene was shown to increase or decrease in glyphosate treated tobacco callus depending on the concentration of available IAA (Lee and Dumas, 1983). Some phytohormones are known to act antagonistically, such as JA and SA, which are major players in the induction of chemical defenses in plants (Robert-Seilaniantz et al., 2011; Thaler et al., 2012). Thus, by affecting the production of a single phytohormone, GBH residues in soil may indirectly alter other phytohormones and potentially plant responses to a magnitude of abiotic and biotic environmental stressors, such as pathogen infection and herbivory (Gomes et al., 2014; Fuchs et al., 2021). We assume that glyphosate-modulated physiological processes in plants stimulate a diverse range of chemical signaling and cross-talk, which needs to be addressed in future studies (indicated in Figure 1). Furthermore, recent studies suggest that moderate stressors in early stages of plant development can act as priming cues, which stimulate plant metabolism in order to overcome unfavorable environmental conditions (Kerchev et al., 2020; Paim et al., 2020). Our result that GBH residues in soil promoted ABA production in potatoes together with increased biomass indicates that GBH residues may be perceived as such a moderate stressor.

### The Effect of Phosphate Addition on Phytohormone Pools

Phosphate treatment alone significantly affected auxins and CKs predominantly in strawberries, but in combination with GBH, phosphate treatment did not alter strawberry plant response on hormonal level compared to control treatment. In strawberry plants, phosphate treatment induced the levels of the growth-related hormones CK tZ and IAA. Simultaneously, the concentration of the stress-related hormone ABA was increased. In potato, GBH treatment together with phosphate appeared to enhance the effects of GBH treatment, which indicates a better glyphosate bioavailability (Borggaard and Gimsing, 2008). However, similar concentrations of glyphosate and AMPA found in soils from both treatments (GBH with and without phosphate fertilizer) at the end of the study, indicates that soil properties and weather conditions did not strongly alter leaching or bioavailability of glyphosate (de Jonge et al., 2001).

### Consequences of Glyphosate-Based Herbicide-Altered Phytohormone Pools to Plant Defense

In contrast to our hypothesis that GBH treatment decreases plant resistance to herbivores leading to an increase in the amount of damage, we detected less herbivore damage on oat plants growing in GBH-treated soils. Less leaf damage of GBH exposed oat plants indicates that plants may be better defended. Phytohormones play a key regulatory function in induced plant defenses (Berens et al., 2017). However, none of the phytohormones, which are commonly involved in plant defense, i.e., ABA, JA, or SA were markedly increased by GBH soil treatment in oat. Instead, the content of shikimate-derived compounds, such as the auxins PAA and IAA, as well as BzA were lower in plants growing in GBH-contaminated soils compared to the controls. BzA is a general precursor for many phenolic compounds, which are responsible for many plant functions (Widhalm and Dudareva, 2015). It is, however, notable that some defense-related compounds such as condensed tannins are derived from the shikimate pathway prior to the target site of glyphosate and they accumulated in plants exposed to sub-lethal glyphosate doses (Ossipov et al., 2003). The accumulation of condensed tannins can cause an increase in plant defense, which could explain the detected low levels of herbivore damage in oat, when growing in glyphosate-treated soil (Hammerschmidt, 2018). On the other hand, plants may be less appealing for herbivorous insects, which can be mediated by changes in olfactory cues of plants (Arimura et al., 2009). The emission of volatile organic compounds can be affected by low glyphosate doses (D’Alessandro et al., 2006). Volatile organic compounds often serve as a foraging cue for insect herbivores and altered concentrations or compositions can increase or reduce the attraction of herbivorous insects and their predators (Gatehouse, 2002; Fuchs and Krauss, 2019), which may explain the differences in herbivore damage in oat plants. These results provide first indications that glyphosate-mediated changes in plant resistance to herbivores can be far more complex than suppression of defense-related phytohormones in plants.

## Conclusion

Our results emphasize the importance of understanding the role of herbicide residues in soil on crop plants. Here we demonstrated the effects of GBH residues in soil on hormonal homeostasis and performance of non-target crop plants, as well as indicated interactions with herbivores. Accordingly, the consequences to non-target plants can range from growth stimulating to changes with their biotic environment. Herbicide residues are ubiquitous and it is necessary to unravel their consequences for ecological interactions and their involvement in shaping evolutionary processes (Riedo et al., 2021). In conclusion, to elucidate the full picture of effects of GBH residues, it requires thorough understanding of the “soil legacy” including the study of soil microbiota and how it is affected by persistent herbicide use.

## Data Availability Statement

The raw data supporting the conclusions of this article will be made available by the authors, without undue reservation.

## Author Contributions

BF, MH, KS, and AM: conception and design of the study. ML, AM, RV, and PD: data collection. PD and BF: data analysis. BF, ML, and AM: manuscript drafting. BF, MH, KS, AM, ML, and RV: manuscript revision for critical intellectual content. BF: writing the final version of the manuscript. All authors read and approved the final manuscript.

## Conflict of Interest

The authors declare that the research was conducted in the absence of any commercial or financial relationships that could be construed as a potential conflict of interest.

## Publisher’s Note

All claims expressed in this article are solely those of the authors and do not necessarily represent those of their affiliated organizations, or those of the publisher, the editors and the reviewers. Any product that may be evaluated in this article, or claim that may be made by its manufacturer, is not guaranteed or endorsed by the publisher.

## Footnotes

1
http://eurofins.fi/

2
https://agrocontrol.nl/en/home-en/

## Supplementary Material

Supplementary Table 1 Mean ± S.E. concentrations of analyzed metabolites in all plants per treatment.

Supplementary Table 2 ANOVA and *post hoc* test results from treatment comparisons per plant species.

## Abbreviations

ABA: abscisic acid
ACC: 1-Aminocyclopropane-1-carboxylic acid
AMPA: aminomethylphosphonic acid
BzA: benzoic acid
CK: cytokinin
ET: ethylene
GBH: glyphosate-based herbicide
IAA: indole-3-acetic acid
IAM: indole-3-acetamide
JA: jasmonic acid
OxIAA: 2-Oxindole-3-acetic acid
PA: phaseic acid
PAA: phenylacetic acid
SA: salicylic acid.
