# Supplementary material for: A Glyphosate-Based Herbicide in Soil Differentially Affects Hormonal Homeostasis and Performance of Non-target Crop Plants
Source: Front Plant Sci. 2022 Jan 27;12:787958. doi: 10.3389/fpls.2021.787958 (PMC8829137; doi:10.3389/fpls.2021.787958)
Supplement: Supplementary Table 1 — Mean ± S.E. concentrations of analyzed metabolites in all plants per treatment. [file Data_Sheet_1.docx]

**Supporting information**

Table S1 The effect of different treatments (P, G, PG) on the content of phytohormones and their metabolites in leaves of in oat, potato and strawberry. Mean ± S.E. in pmol g^-1^ fresh weight are shown. Significant differences by Dunnett’s Posthoc test are highlighted in Figure 1, 2 and Table S2. N=40

|  | Oat | | | | Potato | | | | Strawberry | | | |
| --- | --- | --- | --- | --- | --- | --- | --- | --- | --- | --- | --- | --- |
|  | C | P | G | PG | C | P | G | PG | C | P | G | PG |
| BzA | 8010.6±718.6 | 5147.4±319.8 | 3849.8±343.3 | 3868.4±749.0 | 3743.0±574.0 | 3856.2±689.5 | 4117.7±1151.3 | 2801.4±268.1 | 5593.6±628.2 | 4232.6±513.3 | 6500.3±950.8 | 6283.8±959.2 |
| SA | 1007.2±198.7 | 668.6±57.4 | 667.9±60.7 | 738.0±65.7 | 911.8±128.5 | 1851.8±286.0 | 1363.0±212.7 | 1043.7±104.2 | 18837±1854.1 | 12445±1205.2 | 13614±1625.3 | 14024±1824.8 |
| IAA | 72.2±6.4 | 77.8±10.5 | 43.6±3.2 | 36.2±3.9 | 36.4±4.0 | 45.0±8.4 | 66.2±7.7 | 47.7±8.6 | 22.0±2.5 | 48.0±9.9 | 22.6±4.4 | 27.8±4.5 |
| PAA | 368.8±47.4 | 337.2±30.8 | 127.3±8.6 | 244.3±38.9 | 1941.7±256.2 | 1647.2±243.8 | 3317.8±726.5 | 3804.8±519.0 | 1336.0±165.4 | 1094.2±197.9 | 435.5±48.3 | 1007.0±214.4 |
| IAM | 43.4±7.9 | 24.6±3.0 | 30.9±3.7 | 15.0±1.4 | 909.8±365.4 | 477.8±123.0 | 501.9±121.6 | 679.7±89.9 | 370.6±54.4 | 499.8±109.7 | 303.5±47.2 | 462.5±102.9 |
| OxIAA | 13.3±3.8 | 19.4±2.9 | 4.3±1.3 | 20.7±5.2 | 320.9±65.6 | 330.3±58.4 | 335.2±50.8 | 247.9±39.0 | 537.6±80.2 | 630.2±206.9 | 496.3±114.5 | 846.4±223.6 |
| ABA | 19.2±2.8 | 11.9±2.1 | 19.5±2.0 | 14.6±1.1 | 51.5±7.0 | 50.1±5.4 | 97.4±21.2 | 58.6±9.8 | 765.9±81.7 | 1101.7±122.0 | 770.7±70.1 | 881.4±98.1 |
| PA | 62.4±14.0 | 45.7±4.5 | 41.2±6.4 | 22.2±3.4 | 121.7±28.2 | 60.6±10.8 | 76.0±16.7 | 43.5±9.4 | 12.2±2.5 | 15.2±3.2 | 26.4±3.9 | 26.8±5.8 |
| JA | 3.6±0.7 | 5.3±0.8 | 3.0±0.5 | 3.1±0.5 | 9.9±1.7 | 15.9±3.3 | 42.1±6.4 | 14.6±2.3 | 60.8±9.8 | 40.1±7.1 | 50.8±7.3 | 58.8±10.2 |
| ACC | 20.0±1.6 | 18.9±2.3 | 29.9±6.6 | 16.0±1.8 | 7.5±0.9 | 6.1±0.8 | 10.8±1.7 | 7.3±1.0 | 3.3±0.6 | 2.8±0.6 | 2.4±0.5 | 3.9±0.8 |
| CK_tZ | 25.6±2.2 | 41.1±5.2 | 29.0±4.2 | 26.0±2.8 | NA | NA | NA | NA | 4.4±1.1 | 8.2±1.4 | 2.1±0.6 | 2.0±0.9 |
| CK_DZ | 1.7±0.3 | 1.5±0.4 | 4.6±0.6 | 2.5±0.6 | 3.9±0.6 | 3.0±0.5 | 4.2±0.8 | 2.1±0.3 | 6.3±1.2 | 4.2±0.8 | 1.7±0.6 | 6.0±1.4 |
| CK_iP | NA | NA | NA | NA | NA | NA | NA | NA | NA | NA | NA | NA |
| CK_cZ | 8.0±0.9 | 5.2±0.6 | 6.4±0.7 | 9.8±1.0 | NA | NA | NA | NA | NA | NA | NA | NA |
| CKR | 15.2±1.4 | 16.2±1.3 | 22.9±2.2 | 13.9±2.0 | 8.8±1.1 | 7.0±0.8 | 4.5±0.6 | 5.1±0.9 | 4.7±0.8 | 6.7±0.5 | 4.7±0.7 | 4.7±0.9 |
| CKN | 61.6±6.7 | 104.1±17.9 | 75.3±8.9 | 68.1±7.9 | 1014.9±48.0 | 1019.0±58.5 | 1012.5±72.1 | 962.5±64.7 | 23.4±3.8 | 23.5±3.0 | 25.0±3.6 | 24.9±4.3 |
| CKO | 327.5±23.0 | 364.4±20.9 | 370.1±26.2 | 304.9±10.4 | 32.5±5.8 | 44.8±7.3 | 9.6±1.6 | 16.1±2.7 | 108.9±24.9 | 30.7±1.8 | 103.1±15.6 | 99.6±13.3 |

Abbreviations: BzA, Benzoic acid; SA, Salicylic acid; PAA, Phenylacetic acid; IAM, Indole-3-acetamide; IAA, Indole-3-acetic acid; OxIAA, 2-Oxindole-3-acetic acid; ABA, Abscisic acid; PA, Phaseic acid; ACC, 1-Aminocyclopropane-1-carboxylic acid; JA, Jasmonic acid; CK bases, sum of active cytokinin bases (DZ, dihydrozeatin; tZ, *trans*-zeatin; iP, isopentenyladenine; cZ, *cis*-zeatin); _R, ribosides; _O, O-glycosides; _N, N-glycosides;

Table S2 F-statistic from one way ANOVA and Posthoc test (glht) comparison of treatments to control. p < 0.05 in bold, NA where no data available

| **Oat** | | | | | | |
| --- | --- | --- | --- | --- | --- | --- |
| **ANOVA (treatment C, P, PG)** | | | | Glht (p-value) | | |
| ***Phyto-hormone*** | F | df | p | P-C | G-C | PG-C |
| **BzA** | 11.815 | 36 | **<0.001** | **0.003** | **<0.001** | **<0.001** |
| **SA** | 2.049 | 36 | 0.124 | 0.101 | 0.101 | 0.233 |
| **IAA** | 9.146 | 34 | **<0.001** | 0.888 | **0.011** | **0.002** |
| **PAA** | 9.85 | 36 | **<0.001** | 0.856 | **<0.001** | **0.04** |
| **IAM** | 5.954 | 32 | **0.002** | **0.028** | 0.171 | **<0.001** |
| **OxIAA** | 4.171 | 32 | **0.013** | 0.522 | 0.194 | 0.375 |
| **ABA** | 2.943 | 33 | **0.047** | 0.064 | 0.998 | 0.334 |
| **PA** | 3.668 | 34 | **0.022** | 0.394 | 0.199 | **0.006** |
| **JA** | 3.018 | 34 | **0.043** | 0.146 | 0.786 | 0.868 |
| **ACC** | 2.68 | 36 | 0.061 | 0.994 | 0.158 | 0.79 |
| **tZ** | 3.765 | 36 | **0.019** | **0.016** | 0.864 | 0.999 |
| **DZ** | 7.705 | 36 | **<0.001** | 0.098 | **<0.001** | 0.563 |
| **cZ** | 5.908 | 36 | **0.002** | 0.052 | 0.37 | 0.312 |
| **CK_R** | 5.332 | 36 | **0.004** | 0.945 | **0.009** | 0.918 |
| **CK_N** | 2.769 | 36 | 0.056 | **0.03** | 0.723 | 0.957 |
| **CK_O** | 2.19 | 36 | 0.106 | 0.466 | 0.352 | 0.786 |
| **Potato** | | | | | | |
| **ANOVA (treatment C, P, PG)** | | | | Glht (p-value) | | |
| ***Phyto-hormone*** | F | df | p | P-C | G-C | PG-C |
| **BzA** | 0.598 | 36 | 0.62 | 0.999 | 0.97 | 0.699 |
| **SA** | 4.53 | 36 | **0.009** | **0.005** | 0.261 | 0.934 |
| **IAA** | 2.71 | 35 | 0.06 | 0.763 | **0.025** | 0.598 |
| **PAA** | 4.748 | 36 | **0.006** | 0.948 | 0.124 | **0.025** |
| **IAM** | 0.905 | 33 | 0.449 | 0.328 | 0.372 | 0.777 |
| **OxIAA** | 0.572 | 35 | 0.638 | 0.999 | 0.996 | 0.67 |
| **ABA** | 3.088 | 35 | **0.04** | 0.999 | **0.044** | 0.96 |
| **PA** | 3.164 | 32 | **0.038** | 0.071 | 0.202 | **0.014** |
| **JA** | 13.344 | 34 | **<0.001** | 0.598 | **<0.001** | 0.759 |
| **ACC** | 3.097 | 36 | **0.039** | 0.737 | 0.116 | 0.999 |
| **tZ** | NA | NA | NA | / | / | / |
| **DZ** | 2.641 | 36 | 0.064 | 0.555 | 0.967 | 0.092 |
| **cZ** | NA | NA | NA | / | / | / |
| **CK_R** | 5.128 | 36 | **0.005** | 0.335 | **0.003** | **0.012** |
| **CK_N** | 0.188 | 36 | 0.904 | 1 | 1 | 0.878 |
| **CK_O** | 10.55 | 36 | **<0.001** | 0.199 | **0.006** | 0.062 |
| **Strawberry** | | | | | | |
| **ANOVA (treatment C, P, PG)** | | | | Glht (p-value) | | |
| ***Phyto-hormone*** | F | df | p | P-C | G-C | PG-C |
| **BzA** | 1.685 | 36 | 0.188 | 0.481 | 0.755 | 0.87 |
| **SA** | 2.719 | 32 | **0.061** | **0.03** | 0.114 | 0.14 |
| **IAA** | 3.593 | 31 | **0.025** | **0.018** | 0.999 | 0.831 |
| **PAA** | 5.07 | 36 | **0.005** | 0.623 | **0.002** | 0.388 |
| **IAM** | 1.141 | 35 | 0.346 | 0.56 | 0.893 | 0.788 |
| **OxIAA** | 0.766 | 30 | 0.522 | 0.971 | 0.997 | 0.495 |
| **ABA** | 2.738 | 36 | 0.058 | **0.045** | 1 | 0.724 |
| **PA** | 1.916 | 22 | 0.156 | 0.963 | 0.196 | 0.165 |
| **JA** | 1.159 | 36 | 0.339 | 0.239 | 0.757 | 0.997 |
| **ACC** | 3.097 | 36 | **0.356** | 0.934 | 0.637 | 0.784 |
| **tZ** | 7.989 | 36 | **<0.001** | **0.034** | 0.279 | 0.245 |
| **DZ** | 4.16 | 36 | **0.013** | 0.362 | **0.009** | 0.991 |
| **cZ** | NA | NA | NA | / | / | / |
| **CK_R** | 1.68 | 36 | 0.189 | 0.187 | 1 | 1 |
| **CK_N** | 0.051 | 36 | 0.984 | 1 | 0.982 | 0.986 |
| **CK_O** | 5.185 | 36 | **0.004** | **0.005** | 0.989 | 0.957 |
